# Genomic predictors of radiation response: recent progress towards personalized radiotherapy for brain metastases

**DOI:** 10.1038/s41420-024-02270-2

**Published:** 2024-12-18

**Authors:** Paul M. Harary, Sanjeeth Rajaram, Maggie S. Chen, Yusuke S. Hori, David J. Park, Steven D. Chang

**Affiliations:** 1 Department of Neurosurgery, Stanford University School of Medicine, Stanford, CA USA; 2 Medical Scientist Training Program, Stanford University School of Medicine, Stanford, CA USA

**Keywords:** CNS cancer, Experimental models of disease

## Abstract

Radiotherapy remains a key treatment modality for both primary and metastatic brain tumors. Significant technological advances in precision radiotherapy, such as stereotactic radiosurgery and intensity-modulated radiotherapy, have contributed to improved clinical outcomes. Notably, however, molecular genetics is not yet widely used to inform brain radiotherapy treatment. By comparison, genetic testing now plays a significant role in guiding targeted therapies and immunotherapies, particularly for brain metastases (BM) of lung cancer, breast cancer, and melanoma. Given increasing evidence of the importance of tumor genetics to radiation response, this may represent a currently under-utilized means of enhancing treatment outcomes. In addition, recent studies have shown potentially actionable mutations in BM which are not present in the primary tumor. Overall, this suggests that further investigation into the pathways mediating radiation response variability is warranted. Here, we provide an overview of key mechanisms implicated in BM radiation resistance, including intrinsic and acquired resistance and intratumoral heterogeneity. We then discuss advances in tumor sampling methods, such as a collection of cell-free DNA and RNA, as well as progress in genomic analysis. We further consider how these tools may be applied to provide personalized radiotherapy for BM, including patient stratification, detection of radiotoxicity, and use of radiosensitization agents. In addition, we describe recent developments in preclinical models of BM and consider their relevance to investigating radiation response. Given the increase in clinical trials evaluating the combination of radiotherapy and targeted therapies, as well as the rising incidence of BM, it is essential to develop genomically informed approaches to enhance radiation response.

## Facts

- Radiotherapy is a key treatment approach for brain metastases, with increasing use of precision modalities such as stereotactic radiotherapy
- While precision radiotherapy has improved outcomes, there remains significant variability in tumor and patient radiation response that cannot be accounted for by lesion type, histology, size, or anatomic features alone
- Molecular alterations appear to be a major contributor to radiation response, with recent progress in the identification of specific genetic predictors of radiation treatment outcomes
- Increasing use of genomic profiling in radiotherapy for brain metastases is anticipated, particularly for treatment planning, response monitoring, and selection of targeted therapies

## Open questions

- Can genomic profiling of brain metastases be used to optimize radiotherapy treatment planning to reduce serious adverse effects?
- How can genomic biomarkers be used in treatment response monitoring to support adaptive radiotherapy approaches?
- Can targeted therapies be used to sensitize radioresistant lesions?
- What role will preclinical models play in the identification of additional genomic predictors of radiation response?

## Introduction

Recent progress in targeted systemic therapies for solid tumors has contributed to significantly increased survival times for patients with metastatic disease. However, this progress has been accompanied by a higher incidence of brain metastases (BM), with an estimated 10-40% of primary cancers developing intracranial involvement [1, 2]. Notably, BM occurs tenfold more frequently than primary brain tumors [3]. Given that the blood-brain barrier (BBB) excludes many systemic therapies, local control approaches such as surgical resection are used for initial management, often in combination with radiotherapy [4]. In particular, high-precision radiotherapy techniques such as stereotactic radiosurgery (SRS) have recently been shown to be effective as first-line treatments for many central nervous system (CNS) metastases [1, 5]. Importantly, these methods have fewer neurotoxic effects than traditional whole-brain radiation therapy (WBRT), therefore leading to less cognitive deterioration [5, 6]. Furthermore, increasingly sophisticated dose fractionation and staging protocols now enable the management of a large number of BM with SRS, with ongoing clinical trials evaluating the treatment of up to 20 lesions [7].

Despite these significant advances, current radiotherapy approaches for BM have several meaningful limitations. An estimated 10–20% of BM develop local progression following SRS treatment [8, 9]. For patients with disease recurrence post-SRS, an increasingly frequent scenario given the rise in BM incidence, the ideal treatment approach remains unclear [10]. Future studies, including molecular profiling, may be needed to determine which patients may be the best candidates for multiple SRS courses. In addition, focal radiation strategies such as SRS present a significant risk of distant failure, with new BM arising likely due to residual clusters of tumor cells which were not eliminated by initial radiation treatment. A study of 112 patients treated with SRS reported a 1-year distant intracranial failure rate of 54%, with tumor histology and number of BM as significant predictors [11]. Furthermore, serious adverse effects of radiation, although rare, remain a meaningful risk [12]. Radiation necrosis (RN) may occur from months to years following treatment, affecting a reported 3–24% of patients [13]. Notably, there is significant interpatient variability in radiotherapy adverse events despite similar dosimetry. As gene sequencing technologies have become more accessible and scalable, emerging evidence suggests that genetic variation may account for a meaningful degree of heterogeneity in treatment response [14]. Nevertheless, radiotherapy treatment guidelines remain predicated on uniform population-level radiation response [15]. This suggests an opportunity for genomically informed radiotherapy planning, with treatment recommendations reflecting the wide spectrum of disease.

Radiotherapy is particularly ineffective in controlling BM from “radioresistant” primary tumors, such as melanoma, renal cell carcinoma, and sarcoma [1, 16]. Tumor radioresistance, in which there is limited cell death following exposure to therapeutic ionizing radiation, is thought to result from several distinct mechanisms, including the proliferation of cancer stem cells (CSCs) leading to tumor regrowth following treatment [17]. While SRS has been shown to be superior to WBRT for the treatment of radioresistant histologies, the persistence of previously irradiated BM remains a significant contributor to morbidity and mortality [6, 18]. Current strategies for overcoming BM radioresistance primarily concern tuning of physical parameters of radiotherapy, such as dose fractionation, rather than using biologically targeted approaches. However, genomically informed pharmacological treatment has recently shown promise for radioresistant BM [14].

Furthermore, radiotherapy treatment outcomes are currently evaluated from volumetric and diameter measurements on magnetic resonance imaging (MRI). Such changes are comparatively slow to appear, often lagging behind biological response by months [19, 20]. This low detection speed means that determination of radiation response, and any necessary treatment adjustments, are similarly delayed. Alternative predictive biomarkers have been the subject of growing investigation. Notably, tissue sampling BM poses unique challenges associated with the relative inaccessibility of the brain. For example, when surgical resection is not indicated, then genetic material cannot be collected using conventional tissue-based approaches. Recent advances in the field of liquid biopsy have opened novel avenues for less invasive acquisition of genetic material.

In this review, we discuss key molecular pathways implicated in radioresistance, advances in tumor genomic sampling methods, and applications to personalized radiotherapy. We specifically explore how genomic testing may be used in three primary radiotherapy applications for BM: patient stratification, early radiation treatment response monitoring, and selection of systemic therapies for use in combination with radiotherapy. Recent reports have highlighted the use of blood biomarkers to determine the extent of BM and predict survival outcomes [21, 22]. The importance of rational selection of immunotherapy to augment BM treatment response has similarly been reviewed [23]. By comparison, we aim to describe recent progress in genomic profiling to guide radiotherapy for BM, with an emphasis on bench-to-bedside implementation. To this end, we additionally consider progress in preclinical models of BM, including brain organoid and xenograft models.

## Key mechanisms of radioresistance in brain metastases

Ionizing radiation induces cytotoxicity by generating DNA damage in exposed cells. Sufficient damage can overcome the ability of target cells to recover, causing cell death through interphase apoptosis or, more commonly, mitotic catastrophe [24]. This DNA damage occurs through two routes: direct DNA strand breaks, and indirect DNA modification through the generation of free radicals. The intrinsic ability of cancer cells to tolerate and recover from this DNA damage can stem from several pathways, including anti-apoptotic mechanisms, cell cycle arrest, DNA damage repair (DDR) and nucleotide metabolism, the oxidative/electrophilic stress response to free radicals, autophagy. Additionally, features of the tumor microenvironment, such as hypoxia and immune tolerance, may be protective against free radical generation and immune clearance, respectively.

Monteiro et al. further investigated the contribution of the tumor microenvironment to radioresistance in BM [14]. Using in vivo experimental models, they revealed that astrocyte-derived cytokines may induce secretion of S100A9 by cancer cells, thereby activating the RAGE pathway and resulting in radioresistance in an NF-kB/JunB-dependent manner. Additionally, it was found that S100A9 levels, as determined by liquid biopsy, were a significant predictor of radiation response in a cohort of brain-metastatic NSCLC, breast cancer, and melanoma patients. Notably, pharmacological inhibitors of S100A9 are permeable to the BBB [14]. While further investigation may reveal the genetic basis for the heterogeneity in S100A9 secretion, this report presents a robust example of the potential of liquid biopsy in personalized radiotherapy.

Factors intrinsic to the primary cancer are also known to contribute to radioresistance. Binkley et al. analyzed mutations in 232 genetically-profiled NSCLC patients and found that about half of the local recurrences, post-radiotherapy occurred in tumors with *KEAP1/NFE2L2* mutations, indicating these mutations are major drivers of clinical radioresistance [25]. NFE2L2, also known as NRF2, is a transcription factor involved in cellular response to oxidative stress. KEAP1 is a ubiquitin adapter for NFE2L2 that normally targets the protein for constitutive degradation. Ionizing radiation causes oxidative stress, which oxidizes KEAP1, inducing a conformational change and releasing NFE2L2 [26]. Notably, all reported mutations in *NFE2L2* were gain-of-function, and 40% in *KEAP1* were loss-of-function, suggesting that constitutive antioxidant pathway stimulation is protective against ionizing radiation. Critically, the study also demonstrated that glutaminase inhibition may preferentially radiosensitize *KEAP1*-mutant cells through the depletion of glutathione, a key endogenous antioxidant. The utility of *KEAP1/NFE2L2* mutations for the prediction of radiosensitivity demonstrates the importance of genomic profiling for primary and metastatic lung cancer. Furthermore, *KEAP1* mutations have been associated with radioresistance in multiple cancer types, including prostate cancer [27], head and neck squamous cell carcinoma [28], and meningioma [29, 30].

Current personalized radiogenomics approaches for all indications include a 10-gene signature for radiosensitivity, which includes expression scores for *AR*, *JUN*, *STAT1*, *PKCB*, *RELA*, *ABL1*, *SUMO1*, *PAK2*, *HDAC1*, and *IRF1* [31]. These genes have diverse functions that reflect the breadth of biological mechanisms implicated in radioresistance, ranging from NF-kB signaling, to protein modification, epigenetic DNA alteration, and interferon response, among others.

## Advances in tumor sampling methods and genomic analysis

Progress in genomic profiling of BM has been supported by advances in two key domains. First, increasingly affordable and sophisticated methods have been developed for sampling tumor genetic material, including both tissue-based and liquid biopsy approaches. In particular, liquid biopsy holds great promise due to its minimally invasive nature, relative speed, and potential to capture composite information from both primary tumor and BM [32]. Second, analytical tools and frameworks for interpreting the actionability of genomic results are rapidly evolving. Here, we discuss developments in these two areas, with a focus on liquid biopsy of BM and rational interpretation of variants.

### Sampling brain metastases

Historically, prognostic models for BM, such as recursive partitioning analysis (RPA) [33] and Graded Prognostic Assessment (GPA) [34], have incorporated tumor histology as a factor, typically based on biopsy of the primary tumor. Similarly, histologic grade has been used to inform systemic pharmacotherapy for BM, in combination with other patient characteristics such as neurological status, radiographic findings, and comorbidities [35]. However, the low survival time following diagnosis of BM has limited the utility of these models [36]. While surgical resection remains a common treatment for solid BM, thereby providing tumor samples for histologic diagnosis and molecular profiling, it is not typically used as a basis for the selection of targeted therapies. In addition, stereotactic biopsy of BM is not part of current practice except in cases of diagnostic uncertainty, such as in the setting of autoimmune disease or immunosuppression, particularly when other metastatic disease is absent [37]. Moreover, solid tissue biopsy is not an option for leptomeningeal disease (LMD). The gold standard for LMD diagnosis, cerebrospinal fluid (CSF) cytology, has a comparatively low sensitivity of 44-67% on single sampling [38]. Consequently, there is a significant need for alternative sampling methods to support genomic profiling of BM, particularly to inform radiotherapy treatment when surgical resection has not been performed.

Liquid biopsy is a minimally invasive approach to cancer diagnosis and monitoring through analysis of circulating tumor cells (CTCs) and tumor-derived factors within blood, CSF, and urine. Genomic profiling of circulating tumor DNA (ctDNA) may additionally reveal actionable variants to inform disease management [39, 40]. Notably, liquid biopsy has the potential to overcome several key limitations of conventional tissue biopsy, which are particularly relevant to CNS radiotherapy. First, solid biopsy is unlikely to capture tumor heterogeneity, either within individual BM or across multiple lesions. This is especially pertinent to possible treatment failure due to expansion of radioresistant clones, which may regrow an irradiated tumor [41, 42]. Second, serial sampling may enable early radiation response monitoring. Treatment planning may then be adapted accordingly in the case of early recurrence or progression. Third, liquid biopsy may be used to non-invasively profile BM when surgery is not indicated, and tissue biopsy cannot be easily obtained. While tissue biopsy remains the standard of care, the above advantages are particularly salient given the increased risk associated with brain biopsy and the more frequent use of radiotherapy alone to manage CNS malignancies.

Current liquid biopsy methods may not provide the same sensitivity or breadth of genomic information as tissue biopsy, particularly in brain cancers. While several recent reports suggest high concordance in guideline-recommended biomarkers between plasma and tissue biopsy [43, 44], this appears to be highest in plasma samples with a large ctDNA tumor fraction. In CSF, however, the fraction of cfDNA has been shown to be higher than in plasma, due to a lower overall quantity of non-tumor DNA [45]. CSF-derived cfDNA has recently been applied in clinical practice for analysis of BM, including being used to guide treatment decisions [46]. Nevertheless, CSF biopsy may fail to capture the full spectrum of relevant alterations, particularly for molecular markers with lower abundance. In addition, lumbar puncture to obtain CSF may not be indicated in cases where BM are small in size and have a low total volume, such that a genetic profile is unlikely to change the treatment approach. For example, SRS has been shown to achieve a 1-year local control rate of >85% for BM smaller than 0.5 cm^3^ [47] and has, therefore, traditionally been recommended for lesions with a maximum diameter of up to 3 cm [15]. Despite these limitations, liquid biopsy remains attractive for personalized radiotherapy due to its minimally invasive and repeatable nature. This may be particularly true for moderate to large lesions, for which radiotherapy response remains variable [48, 49]. In addition, CSF sampling may provide an alternative approach for conditions which are not typically amenable to tissue biopsy, such as leptomeningeal disease (LMD) [50].

Radiomics, in which the features of medical images are used to infer tumor characteristics and predict outcomes, has also demonstrated early potential in identifying BM mutational status. For example, radiomic approaches have been used to determine *EGFR* mutation status in NSCLC BM [51], as well as *BRAF* mutation status in melanoma BM [52]. While this field is still emerging, progress has been rapid, particularly given the increasing use of artificial intelligence [53]. Radiomics may, therefore, represent an additional approach for personalized radiotherapy, which may complement liquid biopsy.

### Identifying clinically relevant biomarkers of radiation response

Several gene mutations have been shown to impact cell survival following radiation. These include germline mutations in *ATM* [54] as well as *BRCA* when poly(ADP-ribose) polymerase inhibition is applied as an adjuvant [55]. Similarly, mutations have been identified that are associated with BM radioresistance. *MET* [56], *TopBP1* [57], and *Claspin* [57], overexpression has been shown to induce radioresistance in patient-derived samples and xenograft-generated radioresistant cells. These mutations are highly relevant to determining the course of treatment for certain patients to limit severe effects of toxicity from sensitization.

Loss-of-function in DNA damage-repair genes has been suggested to be predictive of increased tumor radiosensitivity. Somatic mutations in *ATM*, which encodes a protein kinase which is a key activator of the DNA damage response to double-strand breaks [58], have been associated with a favorable response to radiotherapy [59]. Relatedly, mutations in *p53* have been shown to be associated with radiation response. Specifically in CNS malignancies, loss of p53 has been shown to promote radioresistance in pediatric cell lines of diffuse intrinsic pontine glioma (DIPG) [60], medulloblastoma [61], and neuroblastoma [62]. These findings were consistent with retrospective analyses of DIPG [60] and head and neck cancer patients [63]. Similarly, mutations in *Chk2*, which encodes a kinase downstream of ATM in the ATM/Chk2/p53 pathway, have been reported to contribute to radioresistance in gliomas [64]. Furthermore, Chek1/2 inhibition was shown to reverse radioresistance in CD133-positive glioma stem cells [65]. Notably, DNA methylation signatures in pulmonary adenocarcinoma have recently been reported to be predictive of BM development, suggesting the need for more frequent surveillance imaging and possibly more intense irradiation of the primary tumor [66].

One difficulty of genetically mapping BM is the heterogeneity within patient samples from different sites. Whole-exome sequencing of matched BM with primary tumors and normal tissue showed that over half of BM maintained clinically relevant genetic alterations from the primary tumor [67]. Driver mutations were found to be mostly clonal, leading to the formation of site-specific metastases with significantly different genetic makeup from the primary site [68, 69]. One hypothesis for the purpose of this metastatic evolution is that BM heterogeneity facilitates immune remodeling in response to immune pressure [69]. Furthermore, such mechanisms may contribute to the formation of cell populations, such as cancer stem cells, which are epigenetically plastic and capable of switching into a migratory, mesenchymal-like state [70]. The migratory abilities of these cells, and their propensity to seed different neural niches, can give rise to the genomic heterogeneity as seen in BM patients [70]. Additionally, BM across several types of cancer appears to have a higher mutational burden than the corresponding primary tumor. It was recently reported that in 53% of BM patients, there were clinically actionable mutations that were not present in the corresponding primary [67]. By comparison, spatially separated BM within an individual patient had generally uniform genetic profiles. This underscores the importance of performing direct molecular characterization of BM, when feasible, as opposed to relying on analysis of the primary tumor alone.

## Personalized radiotherapy for brain metastases

**Fig. 1:**
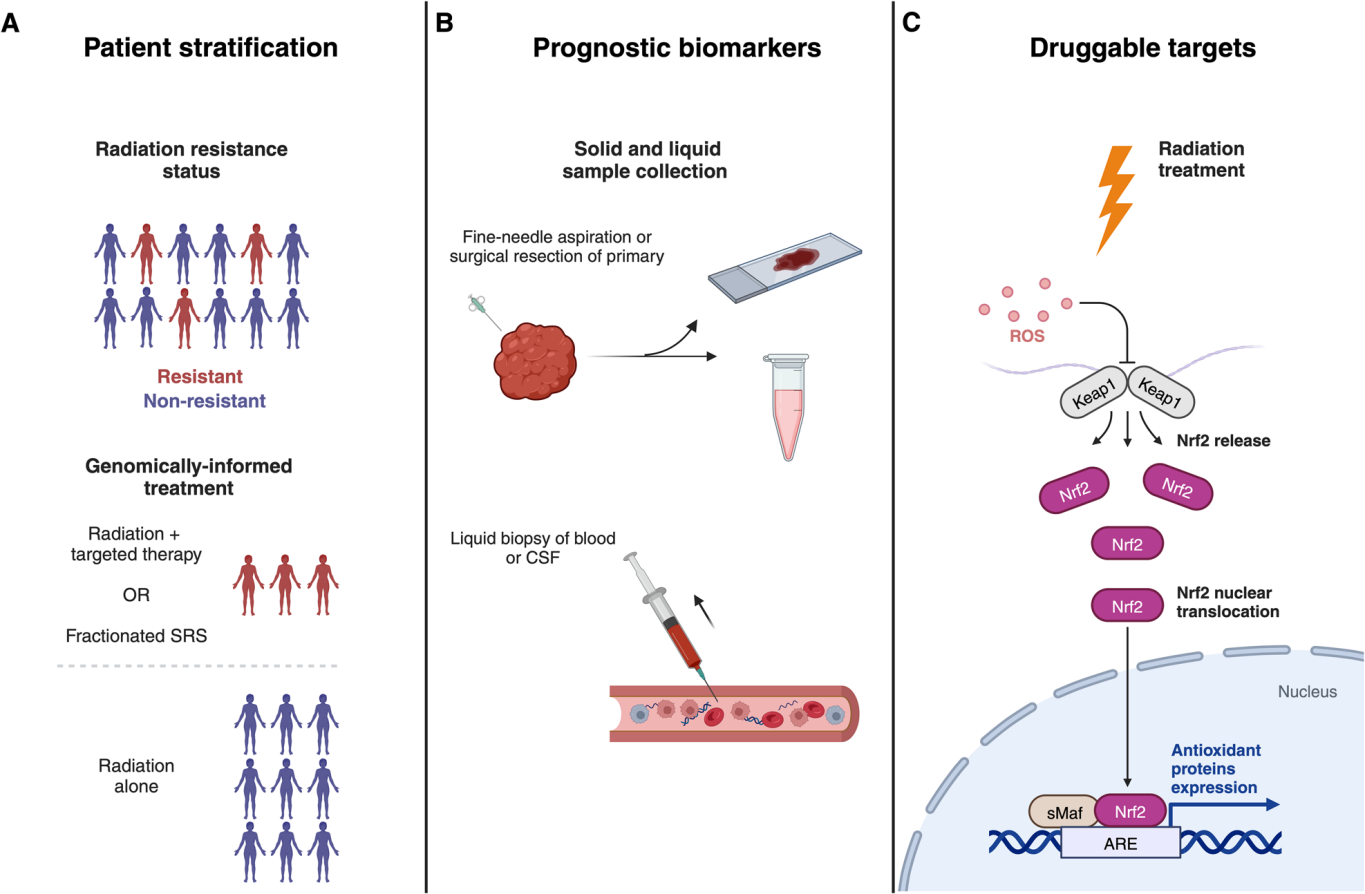
Genomically informed radiotherapy for brain metastases. **A** Genomic profiling of patients with brain metastases may enable the stratification of patients to optimal radiotherapy plan based on genomic alterations which affect radiation response. **B** Genomic alterations may additionally be used as prognostic biomarkers as well as for determination of treatment response, with liquid biopsy having large potential as an alternative to radiographic assessment. **C** Pathway-directed therapies may also be given in combination with radiotherapy to augment patient response. For example, mutations in the KEAP1/NRF2 pathway, which mediates defense against reactive oxygen species (ROS), have been shown to be predictive of local recurrence following radiotherapy and may be targeted to restore radiosensitivity. CSF cerebrospinal fluid, ROS reactive oxygen species, Nrf2 nuclear factor erythroid 2-related factor 2, Keap1 Kelch-like ECH-associated protein, sMaf small Maf proteins; ARE antioxidant response elements.

Genomic profiling has the potential to change the radiation treatment of BM at several different levels of management. While tumor and germline genomic analyses have been widely investigated for use in other cancer treatment modalities, their clinical utility for radiotherapy has been less extensively explored. However, brain tumor radiotherapy is well-suited to benefit from both solid and liquid biopsy, given its role as a primary treatment modality for many brain malignancies. In addition, surgical resection is often performed in combination with radiotherapy, thereby providing solid tissue for BM mutational profiling. Although molecular characterization of resected BM is still in the early stages, it has already provided substantive insights [71, 72]. Non-invasive sampling methods such as liquid biopsy, as described above, are also rapidly progressing as a supplement or alternative to solid biopsy. We contend that genomic profiling may support personalized radiotherapy for BM through patient stratification, treatment response monitoring, and identification of druggable targets to modulate the radiation response (Fig. 1A–C).

### Genomically informed patient stratification

Given the large variability in BM treatment responses, there remains a significant need for clinically relevant biomarkers to prospectively stratify patients to the optimal treatment strategy. Currently, precision radiotherapy is largely based on tumor morphology, size, and number, with minimal reliance on molecular profile [15]. This is in contrast to the use of biomarker status for immunotherapy and targeted therapies in other domains of oncology [73].

Certain genetic alterations have been shown to be highly predictive of radiosensitivity [74, 75]. Patients with germline alterations indicative of increased risk of RN may be poor candidates for SRS. This is further supported by consistency in radiation response across different tissues in an individual [76]. This approach has already been incorporated into radiotoxicity prediction through single nucleotide polymorphism-based models [77]. In addition, BM that are positive for known radioresistance genes may have a higher risk of local recurrence, suggesting the need for more frequent follow-up and possible re-treatment. While prophylactic WBRT remains an area of ongoing debate [78], molecular characterization of tumor radiation response may enable more precise risk-benefit analysis.

The potential to identify BM-specific genetic alterations which are absent in the corresponding primary is a significant advantage of liquid biopsy-based genomic profiling. A recent large parallel genomic analysis of non-small cell lung cancer (NSCLC) BM was performed using primary tumor tissue, BM tissue, plasma, and CSF samples [79]. By comparing liquid biopsy results to those obtained from solid biopsy of BM and the NSCLC primary lesion, Tsakonas et al. were able to identify specific variants which were only present in the CNS compartment. Similarly, Wu et al. examined the efficiency of plasma and CSF ctDNA in detecting NSCLC BM mutations [80]. Plasma and CSF samples were found to contain 83.33 and 27.78% of BM mutations found in solid tissue biopsy, respectively, suggesting that CSF may be a preferred approach for liquid biopsy. Additionally, urine biomarkers have been shown to be predictive of brain tumor disease burden, in both primary glial tumors [81, 82] and other primary CNS malignancies, including meningioma, ependymoma, medulloblastoma, and hemangioblastoma [81]. Overall, however, CSF may have the highest potential to differentiate between mutations in the primary tumor and corresponding BM.

Personalized radiotherapy planning has made significant progress towards translation in the past decade. For example, the genomic adjusted radiation dose (GARD) score was designed to generate a patient-specific schedule of radiation dose and fractionation based on the expression of ten genes in tissue samples [31]. More specifically, the GARD model aims to integrate a genetic radiosensitivity index with the linear quadratic model for radiotherapy [83]. This single value was meant to inform radiation dose adjustment and prognostication, with a higher GARD score suggesting a stronger treatment effect. The GARD model was subsequently validated in additional studies across different cancer types [84–86], including triple-negative breast cancer [84].

Importantly, the clinical relevance of GARD is being increasingly explored through clinical trials. For example, Chiang et al. recently reported their protocol for a phase 3 trial using GARD scoring for treatment personalization in nasopharyngeal cancer [86]. Additionally, an ongoing phase 2 trial is investigating this approach in high-grade soft tissue sarcoma [87]. Notably, soft tissue sarcoma is known to be a radioresistant cancer [88, 89], with a complete response reported to occur in only 8% of patients receiving conventional neoadjuvant radiotherapy [90].

Given continued progress in identifying radiation response genes, expanding the target panel of such models may increase their clinical utility. While other aspects of the tumor microenvironment are likely to impact radiation response, such as hypoxia and neuroanatomical location, molecular profiling may provide significant insight to support individualized radiotherapy planning. However, further clinical validation is needed, particularly for individual gene predictors, given the reliance on retrospective studies.

### Early detection and management of radiotoxicity and disease progression

Despite significant advances in radiation technology and dosing schedules, toxicity remains a common occurrence. Notably, RN is a late effect of SRS, such that treatment has already been delivered at the time it presents with radiographic changes. Moreover, RN is often indistinguishable from pseudoprogression and progression on imaging [91]. A recent systematic review reported pooled estimates of specificity of 72 and 82% for 18-fluorine fluorodeoxyglucose positron emission tomography and gadolinium MRI, respectively, underscoring the limitations of common diagnostic approaches for RN [92]. Genomic biomarkers may provide a more rapid and specific indication of toxicity than radiographic approaches. Bevacizumab is often used to mitigate symptoms of RN through the reduction of nonvascular permeability and associated brain edema [93]. However, early diagnosis of RN remains key to improvement of long-term outcomes. Furthermore, specific biomarkers of RN are needed to avoid unnecessary treatment with bevacizumab, given its significant side effects.

Disease progression following treatment may similarly be monitored through BM genomic profiling. For example, increases in the ctDNA fraction within CSF may be used to identify cancer progression [94]. Since liquid biopsy is repeatable with minimal adverse effects, as opposed to radiological assays, which present a cumulative risk, it may be especially beneficial for patients who require long-term surveillance due to genetic cancer predisposition. In particular, it has been proposed that CSF biopsy may be used to adapt radiotherapy should LMD not respond to other treatments [95]. For example, radiotherapy dose escalation may be performed earlier if ctDNA obtained from CSF does not decrease in concentration [96]. Furthermore, liquid biopsy of plasma and CSF were recently used to compare the evolution of LMD with systemic disease in patients receiving proton craniospinal irradiation (CSI) [97]. It was reported that CSI resulted in alterations of variant allele frequencies that were confined to the CSF, indicating that radiation treatment may result in somatic mutations in LMD that were not present in other tissues. In addition, this suggests that liquid biopsy may provide superior sensitivity for the detection of disease response to CSI.

### Radiosensitization agents—molecular targeting

Biomarkers of radiosensitivity may represent druggable targets for enhancement of radiation response through combination therapy. Radiosensitizers aim to improve the therapeutic index of radiotherapy by preferentially increasing the effects of radiation in tumor cells while sparing adjacent normal tissue [98]. Although many compounds have demonstrated promise at the preclinical stage, the number of radiosensitizers which have shown benefit in patients is highly limited [99, 100]. However, significant mechanistic insight into tumor radiosensitivity has been obtained in the past decade, suggesting that the landscape of radiosensitizers may be changing. In particular, targeted therapies appear to hold great promise. For example, glutaminase inhibition using CB-839 was shown to increase the radiosensitivity of KEAP1/NRF2 mutant NSCLC in an in vitro model [25]. Furthermore, when compared with isogenic wildtype control lines, even a low dose of the inhibitor CB-839 restored the radiation response of the KEAP1/NRF2 mutant cells. Consequently, it was suggested that glutathione depletion may potentiate radiation-induced DNA damage. This was supported by an independent study which reported that CB-839 augmented radiation response in a rodent xenograft model of NSCLC established by injection of a KEAP1-mutant cancer cell line [101]. Such radiosensitization strategies may be especially valuable for medically inoperable patients with radioresistant tumors, for which current treatment options are highly limited.

Several early-phase clinical trials have reported encouraging results for targeted radiosensitizers in CNS malignancies. A phase 1 trial (NCT03423628) of concurrent administration of AZD1390, a selective inhibitor of ATM, and standard of care radiotherapy showed preliminary efficacy for glioblastoma [102]. Notably, the trial included 75 patients with recurrent glioblastoma, out of a total of 115 enrolled, with initial data suggesting increased overall survival relative to the current standard of care. In addition, a phase 2 trial (NCT02655601) investigated the use of BMX-001, an inhibitor of NF-kB and HIF-1α, in combination with radiotherapy and temozolomide in 160 patients with high-grade glioma [103]. This approach was found to increase overall survival, with a median of 31.3 months reported for patients receiving BMX-001 compared with 24.7 months for those treated with radiotherapy and temozolomide alone. Separately, a similar agent has been evaluated for BM in a multi-center phase 1/2 trial (NCT02215512) [104]. RRx-001, a small molecule which promotes the generation of nitric oxide in low-oxygen conditions, was given concurrently with whole-brain radiotherapy to 29 patients with BM, with melanoma and NSCLC as the most common primary tumor types. The drug was found to be well-tolerated, with a favorable treatment response. In addition, two patients with radioresistant BM of melanoma demonstrated partial responses to treatment with RRx-001 and whole-brain radiotherapy, which was highlighted in a separate report by the study authors [105]. Furthermore, a phase 1 trial (NCT02820454) of a gadolinium-based nanoparticle, AGuIX, was assessed in combination with whole-brain radiotherapy for patients with multiple BM [106]. The authors reported a favorable safety profile as well as penetration of the drug into BM from melanoma, lung, breast, and colon cancers, with further trials planned for assessment of efficacy. Additionally, AGuIX is currently being investigated with concurrent radiotherapy and temozolomide in a phase 1/2 trial (NCT04881032) for glioblastoma, with the goal of overcoming radioresistance [107].

Genomically informed therapy may also be used to enhance the radiation-induced antitumor immune response to BM. The abscopal effect describes the regression of distant, unirradiated tumors following local treatment [108]. While abscopal responses (ARs) are thought to be immune-mediated, they remain a rare phenomenon in patients [109]. ARs may hold particular benefits for BM patients and remain an area of ongoing interest for brain and spine malignancies [110]. A significant investigation has been undertaken to elucidate the specific mechanisms underlying ARs and apply combination therapy to improve radiotherapy outcomes, particularly through immune checkpoint inhibitors [111] (Table 1). For example, the landmark phase III PACIFIC trial demonstrated improved overall and progression-free survival when consolidation anti-PD-1 therapy was added to conventional chemoradiation for patients with stage III, unresectable NSCLC, leading this approach to be adopted as a standard of care for this patient population [112]. Despite this early promise, however, attempts to expand the use of combined immune checkpoint blockade and radiotherapy beyond the consolidation setting have had mixed results [113–117]. Notably, concurrent anti-PD-1 therapy and chemoradiation failed to show benefit as upfront (as opposed to consolidation) treatment for stage III, unresectable NSCLC [118]. In addition, a recent trial of combined anti-PD-1 therapy with and without SRS for metastatic head and neck cancer found that this approach neither improved treatment outcomes nor produced an AR [113]. Collectively, this suggests the need for increased molecular profiling of tumors for rational selection of targeted therapies and immunotherapy agents to improve local control of BM, particularly in radioresistant cancers such as melanoma (Table 2). Using biomarker-driven strategies may also support investigation into other promising therapies based on the features of a given tumor and its microenvironment. For example, a key myeloid checkpoint involved in the abscopal effect, CD47, has been shown to be overexpressed in radioresistant tumors from multiple types of primary tumor, including breast cancer [119] and a model of glioblastoma [120]. CD47 blockade has been used to augment radiotherapy response in preclinical models of breast cancer [119] and SCLC [121]. Therefore, genomic profiling may guide the use of CD47-directed therapy in combination with radiation treatment by identifying BM patients who may most benefit. The use of CD47 blockade with radiotherapy for breast cancer BM is already being explored [69, 122].

**Table 1:** Select clinical trials of immune checkpoint inhibition combined with radiotherapy.

Agent class	Clinical trial ID (reference)	Study name (phase)	Year	Tumor type	Radiotherapy modality	Treatment setting	Number of cases (median age, sex)	Outcomes
Anti-PD-1/PD-L1	NCT02105636 [129]	CheckMate-141 (III)	2016	Head and neck squamous cell carcinoma	Prior radiotherapy (91.4%)	Recurrent disease	361 (60, 83.1% M)	Increased median OS with nivolumab (7.5 months) vs. standard therapy (5.1 months)
	NCT02125461 [75]	PACIFIC (III)	2017	Non–small-cell lung cancer	Definitive radiotherapy concurrent with chemotherapy	Consolidation therapy with placebo in patients with stage III NSCLC	713 (64, 70.2% M)	Increased 5-year survival with duvalumab after CRT (42.9%) vs. placebo (33.4%)
	NCT02492568 [130]	PEMBRO-RT (II)	2019	Non–Small Cell Lung Cancer	SBRT	Advanced disease	76 (62, 57.5% M)	Increases in ORR and median PFS were observed with pembrolizumab but did not reach statistical significance
	NCT03807765 [131]	Nivolumab and Stereotactic Radiosurgery for Patients With Breast Cancer Brain Metastases	2021	Breast Cancer Brain Metastases	SRS (post-nivolumab)	Metastatic disease	12 (58, 0% M)	No dose limiting toxicities were reported. 4/12 patients experienced systemic progression during study
	NCT02684253 [76]	Nivolumab With Stereotactic Body Radiotherapy Versus Nivolumab Alone in Metastatic Head and Neck Squamous Cell Carcinoma (II)	2021	Head and neck squamous cell carcinoma	SBRT	Metastatic disease	62 (63, not provided)	No significant difference in ORR, OS, or response duration. Grade 3–5 toxicities were similar.
	NCT02952586 [132]	JAVELIN Head and Neck 100 (III)	2021	Head and neck squamous cell carcinoma	IMRT	Locally advanced disease	697 (60, 83% M)	No significant difference in median PFS (stopped for futility)
	NCT03519971 [81]	PACIFIC-2 trial (III)	2023	Unresectable, stage III NSCLC	Concurrent chemoradiotherapy	Unresectable disease	328 (not provided)	No significant difference in PFS with durvalumab vs. CRT alone
	NCT03040999 [133]	KEYNOTE-412	2024	Head and neck squamous cell carcinoma	Concurrent chemoradiotherapy	Locally advanced disease	804 (58, 82% M)	No significant difference in event-free survival
PD-1 + CTLA4	NCT02320058 [134]	CheckMate-204	2021	Melanoma brain metastases	SRS	Asymptomatic and symptomatic patients	94 (59; 68% M)	3-year response, OS, and median PFS rates were favorable in asymptomatic patients receiving ipilimumab and nivolumab. However, symptomatic patients demonstrated less benefit
	NCT04785287 [135]	Anti-CTLA-4-NF mAb (BMS986218), Nivolumab, and Stereotactic Body Radiation Therapy for the Treatment of Metastatic Solid Malignancies	Ongoing	Solid metastatic cancer	SBRT	Metastatic cancer with ≥ metastatic or primary lesion in bone, adrenal, liver, or lung/chest	13 (not provided)	Ongoing
**Anti-CTLA-4**	NCT00861614 [136]	Ipilimumab versus placebo after radiotherapy in patients with metastatic castration-resistant prostate cancer that had progressed after docetaxel chemotherapy (CA184-043) (III)	2014	Prostate cancer	Bone-directed radiotherapy followed by either ipilimumab or placebo	Metastatic castration-resistant prostate cancer that had progressed after docetaxel chemotherapy	799 (69, 100% M)	No significant difference in OS between ipilimumab and placebo groups, although authors noted that there were signs of drug activity which warrant additional investigation
	NCT02221739 [137]	Radiotherapy induces responses of lung cancer to CTLA-4 blockade (II)	2018	NSCLC	External beam with linear accelerator with IGRT or IMRT	Chemo-refractory metastatic NSCLC	21 (69, 50%); 40/9% with brain metastases	Objective radiographic responses reported in 18% of enrolled patients, with 31% of patients with disease control.
	NCT02239900 [138]	Phase II Trial of Ipilimumab with Stereotactic Radiation Therapy for Metastatic Disease	2019	Metastatic lesions in the liver or lung	Concurrent or sequential stereotactic ablative radiotherapy	Metastatic cancer with at least one metastatic lesion in the liver, lung, or adrenal glands	106 (60, 47% M)	Median PFS time was 2.9 months and median OS time was not reached. An exploratory analysis found that lesions receiving low-dose radiation were more likely to respond than those which were not irradiated

*CTLA-4* cytotoxic T-lymphocyte associated protein 4, *CRT* chemoradiotherapy, IMRT intensity-modulated radiation therapy, *NSCLC* non-small cell lung cancer; *ORR* overall response rate, *OS* overall survival, *PD-1* programmed cell death protein 1, *PD-L1*, programmed cell death-ligand 1, *PFS* progression-free survival, *SBRT* stereotactic body radiation therapy, *SRS* stereotactic radiosurgery, *WBRT* whole-brain radiation therapy.

**Table 2:** Targeted therapy combined with stereotactic radiosurgery for melanoma brain metastases.

Agent class	Author, Year	Treatment stratification	Number of cases by mutation status	Local control rate	Median overall survival
BRAF inhibition	Ly, 2015 [139]	*BRAF* V600E mutation-positive patients received dabrafenib or vemurafenib. Patients with WT BRAF were not treated with BRAF inhibitor therapy	17 patients with *BRAF* mutation, 35 patients with WT *BRAF*	1-year local control rate was significantly increased (*p* = 0.0077) for *BRAF*-mutant (85%) vs. WT *BRAF* BM (51.5%)	No significant difference in OS was found on the basis of *BRAF* mutation or inhibitor therapy status
BRAF inhibition	Ahmed, 2015 [140]	All patients had confirmed *BRAF* mutation	24 patients with *BRAF* V600E mutation	No significant difference in local control compared to chemotherapy alone	Median OS from SRS treatment was 7.2 months
BRAF inhibition	Wolf, 2016 [141]	*BRAF* V600E mutation-positive patients received dabrafenib, vemurafenib, or a combination of the two drugs. Patients with WT *BRAF* were not treated with BRAF inhibitor therapy	35 patients with *BRAF* mutation, 45 patients with WT *BRAF*	Median time to intracranial progression: 3.9 months on BRAF inhibitor, 1.7 months without. Local control rate: 92.5% for all treated tumors, with no difference based on *BRAF* status	Median OS from first SRS procedure: 11.2 months with BRAF inhibitor, 4.5 months for BRAF-WT
BRAF/MEK inhibition	Ahmed, 2015 [140]	All patients had confirmed BRAF mutation	12 patients with *BRAF* mutation	No significant difference in local control compared to chemotherapy alone	BRAF/MEK inhibitor treatment significantly improved OS compared to conventional chemotherapy
BRAF/MEK inhibition	Acharya, 2017 [142]	All patients had confirmed *BRAF* V600E mutation	BRAF/MEK inhibition + SRS: 16, SRS alone: 38	1-year local control rate was 72% for SRS + BRAF/MEK inhibition vs. 66% for SRS alone	OS not specifically reported for BRAF/MEK inhibition cohort

*OS* overall survival, *SRS* stereotactic radiosurgery, *WT* wildtype.

While research in personalized radiotherapy for BM is rapidly developing, significant investigation is still required to validate and translate clinically relevant biomarkers. Therefore, progress in preclinical models of BM represents a central factor in moving genomically informed radiotherapy into the clinic. Importantly, the low translation rate of animal models of BM to human trials has historically been a major obstacle in developing effective therapies [123].

## Preclinical models of brain metastasis radiation response

Animal models have been instrumental in preclinical investigation in radiation therapy, as in many other domains of oncology research. For example, the advantages of dose fractionation were discovered using rams more than a century ago [124]. More recently, rodent models have provided significant insight into the mechanisms of radiobiology, such as the roles of reactive oxygen species and myofibroblasts in radiation-induced fibrosis [125]. However, as the field of cancer biology has revealed the importance of the tumor microenvironment in mediating radiation response, beyond the malignant cells alone [126, 127], the constraints of rodent models have come into sharper focus. For example, despite significant homology between the rodent and human immune systems, there are also meaningful differences [128]. In addition, the tumor vascular supply, which is key in shaping the tumor microenvironment through the delivery of oxygen, nutrients, and immune mediators, may not be fully recapitulated in engineered rodent models of cancer [129, 130]. Furthermore, the tumor microenvironment in these models is often treatment-naïve prior to experimental radiation, which is inconsistent with the majority of radiation therapy patients, particularly those enrolled in clinical trials [131]. These limitations are particularly meaningful when investigating metastatic disease, which does not arise from brain tissue. Here, we consider recent advances in animal models of BM and their applications in investigating genomic determinants of radiation response. While in vitro BM systems are undoubtedly valuable, we limit our discussion to in vivo models, with a focus on progress in patient-derived xenograft and organoid models. We contend that xenotransplantation using such systems, preserving patient genetic background in an in vivo setting, may be well-suited to basic research in personalized radiotherapy.

**Fig. 2:**
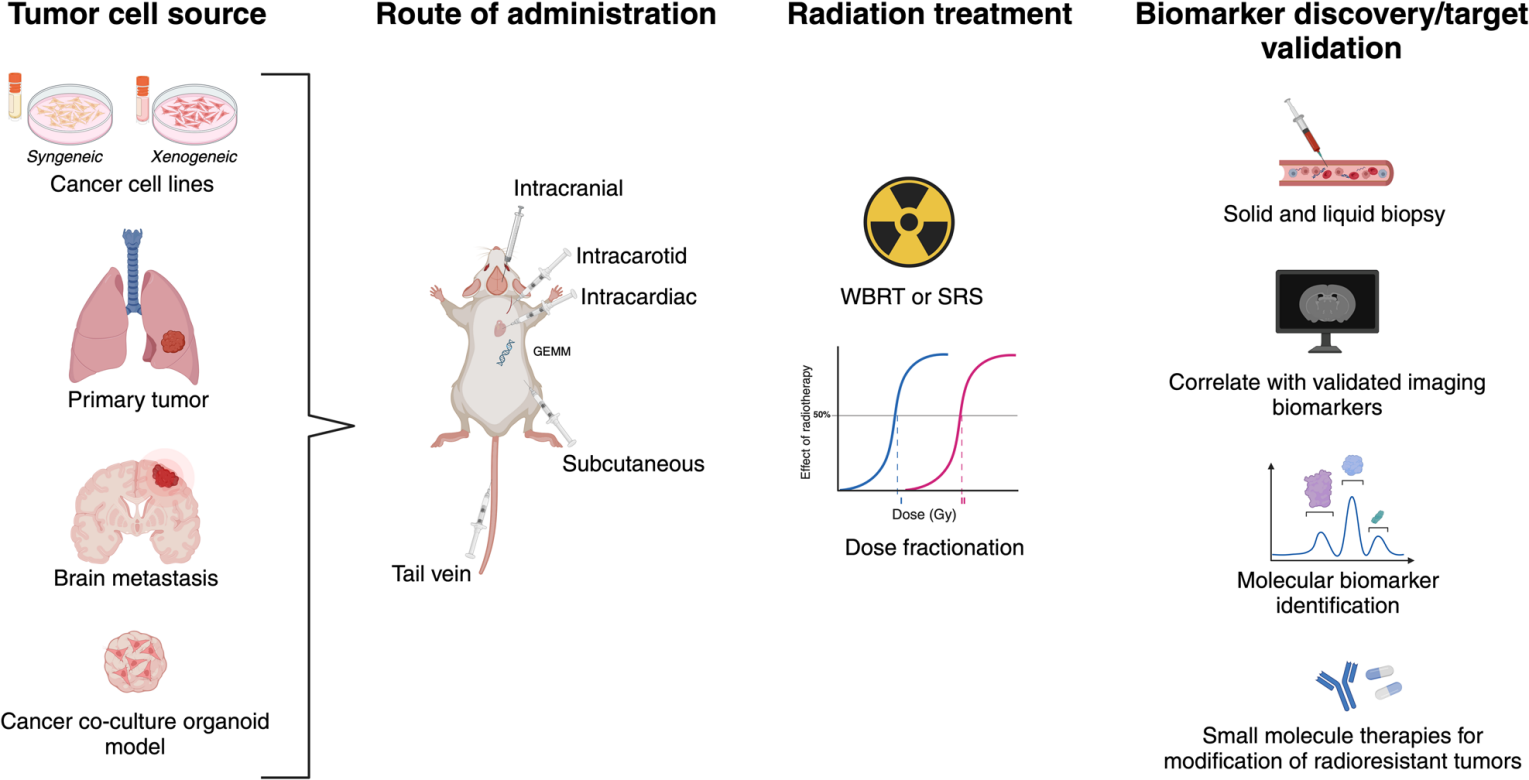
Animal models of brain metastases radiation response. Preclinical models of brain metastases may use cancer cell lines, patient-derived tissue, or co-culture models as the source of tumor cells. These may then be administered through heterotopic or orthotopic transplantation. After engraftment, radiation treatment can be given to model whole-brain radiation therapy (WBRT) or stereotactic radiosurgery (SRS). Subsequent readouts may include transcriptomic, histological, and radiologic data to identify clinically relevant biomarkers of radiation response.

It is essential to consider the specific design needs of an animal model of radiation response by comparison with those for other therapeutic modalities. While no current preclinical model can capture the full complexity and heterogeneity of BM, the selection of a system which is faithful to the most relevant disease aspects may increase predictive value. For example, tumor heterogeneity and molecular profile may be more pertinent to radiation response than full replication of the metastatic process. Key aspects in the design of such a model include the tumor cell source, which may be a cancer cell line, patient-derived primary or metastatic tissue, or three-dimensional co-culture systems (Fig. 2). An additional consideration is the inoculation site, whether ectopic or orthotopic. The modality and parameters of the experimental radiation treatment are similarly important. Finally, the response variables and sample collection methods must be appropriate to the question being investigated.

### Genetically-engineered mouse models

Genetically-engineered mouse models (GEMMs), in which de novo tumors are induced through transgenic knock-in or knockout of target genes, are frequently used to study cancer initiation and progression. Germline gene editing is performed using CRISPR/Cas9 or TALENs, with microinjection of a DNA construct into the zygotic pronucleus or introduction of edited ESCs into a blastocyst [132–135]. In addition, inducible systems such as Cre/loxP and tetracycline-regulated expression can be used to establish tissue-specificity and temporal control over tumor formation [136, 137]. For example, Dickins et al. used a tetracycline-response element to control in vivo expression of a short-hairpin RNA against Trp53 [138], the mouse analog of TP53. This model enabled the authors to investigate the role of TP53 inhibition in the radiation response. A significant advantage of GEMMs is that primary tumors which occur in such models are exposed to a functional immune system and a physiological tumor microenvironment, with subsequent BM occurring spontaneously [139]. In addition, GEMMs do not present issues of interspecies incompatibility, which must be considered in xenograft models.

However, the transgenic approach used in GEMMs presents significant limitations. Most notably, GEMMs demonstrate a low rate of metastatic spread, with animals often succumbing to their primary tumors or extracranial metastases before intracranial disease is seen [140, 141]. Consequently, there are relatively few such models validated for the study of BM, with key primary tumor types such as breast cancer remaining unavailable. Despite the many theoretical advantages of GEMMs, this prevents their more widespread adoption, with allograft and xenograft approaches becoming the main in vivo BM models [142]. Therefore, GEMMs may currently be the least applicable to personalized radiotherapy of the models discussed herein.

### Cancer cell line transplantation

Cancer cell line-derived allograft and xenograft models, in which rodent or human cell lines are introduced by intracardiac, intracarotid, subcutaneous, tail vein, or fat pad injection, remain common platforms in basic oncology research. These approaches are comparatively rapid and widely available. In particular, intracarotid injection has been proposed as a more reliable model of BM due to the high incidence of tumor formation [143]. To enhance the yield of BM in such systems, several cancer cell variants with increased brain-tropism have been established [19, 144, 145]. Recently, Monteiro et al. investigated the S100A9-RAGE-NF-kB-JunB pathway by using intracardiac injection of a breast cancer cell line to establish BM in a mouse model [14]. The authors found that genetic or pharmacological targeting of this pathway was sufficient to reverse radioresistance, leading to increased response to lower doses of radiation.

Cell line xenografts have also been used to model leptomeningeal metastasis from breast and lung cancers [19], which is notable given the complexity of the leptomeningeal microenvironment and the large role of radiotherapy in the management of LMD. However, BM generated through orthotopic transplantation bypasses several steps in the metastatic cascade. Furthermore, tumors generated in cell line-derived xenograft models have been shown to have a comparatively low mutational burden [146, 147], which represents a significant limitation for unbiased genomic profiling. The lack of genetic heterogeneity of cell line-derived xenografts, in particular, has been suggested to be a significant contributor to the low predictive value of these models for human response [148]. To overcome some of these limitations, patient-derived xenografts (PDX) have gained momentum. Nevertheless, cancer cell line transplantation models remain well-suited to certain investigations, particularly when a large number of BM is required.

### Patient-derived xenografts

PDX are established through transplantation of human tumor biopsy samples directly into immunodeficient small animals. This enables the relative conservation of genetic and histological features [149], which is a significant advantage for the development of personalized therapies across several modalities, including radiotherapy. Due to these advantages, PDX are increasing in use, including in drug screening [150, 151]. In a recent landmark study, over 1,000 PDX models across various driver mutations were established for use in drug screening [152]. In the context of BM, PDX may be established using either the primary lesion or corresponding BM. In addition, tumor cells may be introduced into the rodent host through heterotopic or orthotopic transplantation, with the former often performed by subcutaneous injection. For example, breast cancer BM have been modeled through orthotopic injection of the patient primary tumor into mammary fat pad [153]. Important to personalized radiotherapy, PDX may be established from treatment-resistant cancers to more specifically investigate mechanisms of resistance. PDX models have been used to study resistance to cisplatin [154], hormone therapy [155], and BRAF inhibition [151], among others. In addition, Baschnagel et al. used PDX to investigate the role of *MET* in the radiation response [156]. In a model of NSCLC BM with *MET* exon 14 skipping mutation, the authors demonstrated that the application of savolitinib, a selective MET inhibitor, enhanced the effects of radiotherapy. We contend that a similar strategy may be used to generate PDX from radioresistant BM. This may serve as a key resource in biomarker discovery and druggable target validation for genomically informed radiotherapy for BM.

### Transplantation of organoid co-culture systems

A relatively novel approach to modeling brain malignancies has been the co-culture of human brain organoids with cancer cells. Brain organoids are three-dimensional culture models which can be derived from pluripotent stem cells, or from highly proliferative donor tissue such as brain tumors, and more strongly reproduce the structure, function, and cell-type diversity of human brain regions than planar cultures [157, 158]. Furthermore, directed differentiation protocols allow for modeling specific brain regions [159, 160]. Patient-derived glioblastoma organoids have been used to model glioblastoma, allowing for both biobanking and orthotopic transplantation of intact organoids into mice [161]. More recently, brain organoids have been used to study BM through the co-culture of cerebral organoids with dissociated cancer cells [162]. These build upon similar work using ex vivo organotypic slices and 2D cancer cell co-culture [14, 163]. Organoid co-culture has also been used in parallel with a xenograft model to investigate small cell lung cancer (SCLC) BM [164]. Intracranial orthotopic injections of human cancer cells were used to establish SCLC BM in immunodeficient mice. Separately, human cortical organoids were fused with SCLC cells in culture. These complementary approaches revealed the mechanism by which reactive astrocytes are recruited to the tumor microenvironment to promote BM growth. Furthermore, significant progress in the transplantation of cortical organoids into small animal hosts has enabled the in vivo vascularization of these models, as well as providing a degree of functional integrations with host brain [165–167]. We propose that an extension of these model systems—brain organoid technology, cancer cell line co-culture, and organoid transplantation—may be used to more rigorously examine radiation response using human BM cells and neural tissue within an in vivo microenvironment. Given the significant role of the brain tumor microenvironment in radiation response (Table 3), this would represent a key advancement upon current models.

**Table 3:** Features of the brain tumor microenvironment implicated in radioresistance.

Tumor microenvironment component	Function
Damage-associated molecular patterns (DAMPs)	DAMPs are released from irradiated cells and stimulate macrophages to promote an antitumor immune response. Damage to endothelial cells of the blood-brain may result in radiation necrosis, a late complication of radiotherapy.
Immune cell types	Immune components, including microglia, monocytes, monocyte-derived macrophages, T cells, and MDSCs, are key mediators of treatment response and are responsible for the abscopal effect.
Immune checkpoints	Cancer cells may upregulate immune checkpoints such as PD-L1 and CD47, which represent druggable targets for potential enhancement of radiation response.
Reactive astrocytes	Reactive astrocytes have been shown to promote brain tumor progression and may be activated following radiotherapy.
ROS	ROS are generated by cancer cells as well as stromal cells within the tumor microenvironment following treatment with ionizing radiation, promoting upregulation of hypoxia-inducible factor 1a and mounting of an antioxidant defense. ROS may therefore alter the composition and activity of the tumor microenvironment.

*DAMPs* damage-associated molecular patterns, *HIF-1α* hypoxia-inducible factor 1α, *MDSCs* myeloid-derived suppressor cells, *ROS* reactive oxygen species.

### Limitations of preclinical models of radiation response

Small animal models of BM present several meaningful limitations for the study of radiation response. Notably, there are intrinsic differences in human and rodent neural tissue. While the general architecture of the cerebral cortex is conserved across these species, there are discrepancies in cell-type diversity and transcriptional regulation [168, 169], including divergence in gene expression in homologous cell types [170]. Consequently, the response of brain tissue to radiation may differ between rodent preclinical models and human patients, particularly at the molecular level. This may perturb investigation into adverse effects of radiation therapy, such as RN. In addition, current preclinical models are unlikely to fully recapitulate the tumor microenvironment, particularly tumor-immune interactions. Furthermore, immunocompromised animals are commonly used in these experiments, such that the important role of the immune system may not be recapitulated. This is particularly relevant to the study of radiation response, given the role of the immune system in mediating the abscopal effect [108]. While humanized PDX mice have been explored, in which immunodeficient mice are engrafted with human CD45^+^ cells [171], this approach is unlikely to fully capture the human immune response.

Importantly, these preclinical models are resource-intensive and require significant technical expertise to establish and maintain. Tumor xenografts, particularly those using patient-derived tissue, may impose a high cost per experiment and require significant expertise. This is exacerbated in the case of BM, which may have comparatively low engraftment efficiency [156, 172]. While cell culture-based approaches are typically higher throughput, they also require substantial infrastructure, especially for generation and maintenance of pluripotent stem cell-derived organoids [173]. Furthermore, irradiation of small animals and in vitro systems may require specialized equipment and training [174, 175]. Although efforts have been made to standardize radiation doses and delivery methods [14], this remains a significant consideration. Finally, as such models become more sophisticated, concerns about reproducibility across different laboratories may likewise increase.

## Conclusion

Genetic determinants of radiation response appear to be a significant contributor to interpatient variability in current radiation treatment outcomes for BM. While whole-exome sequencing has led to growth in the field of BM molecular genetics, these efforts have not yet been translated to the clinic. Potential applications include patient stratification, high-sensitivity treatment response monitoring, and identification of druggable targets for radiosensitization. These efforts may be supported by non-invasive sample collection methods such as blood and CSF biopsy, as well as more sophisticated genomic analysis. While current liquid biopsy approaches may not provide the same sensitivity or breadth of genomic information as traditional tissue biopsy of BM, significant progress is being made, aided by thoughtful use of preclinical models of BM for genomic biomarker discovery and target validation. Interdisciplinary collaboration will be key to supporting reproducible results, spanning the fields of genetics, neurosurgery, and pharmacology, alongside radiation oncology.
